# Cook County Medical Society

**Published:** 1857-06

**Authors:** 


*Cook County Medical Society.*

At the Annual Meeting of this Society, held April 7, 1857, the following officers were elected for the ensuing year, viz.:—For President, Dr. N. S. Davis; Vice-President, Dr. H. Parker; Secretary and Treasurer, Dr. T. Bevan. Drs. Wagner, J. Newton, E. L. Holmes, and H. F. Smith were elected members. And Drs. Bevan, Wickersham, Woodworth, Peterson, and Cheeney were elected delegates to the State Medical Society; which holds its regular annual meeting in this city, on Tuesday, June 2d, 1857.

